# Prevalence and risk factors associated with asymptomatic malaria among school children: repeated cross-sectional surveys of school children in two ecological zones in Ghana

**DOI:** 10.1186/s12889-021-11714-8

**Published:** 2021-09-17

**Authors:** B. A. Mensah, J. L. Myers-Hansen, E. Obeng Amoako, M. Opoku, B. K. Abuaku, A. Ghansah

**Affiliations:** 1 Department of Epidemiology, College of Health Sciences, Noguchi Memorial Institute for Medical Research, University of Ghana, Accra, Ghana; 2 Department of Parasitology, College of Health Sciences, Noguchi Memorial Institute for Medical Research, University of Ghana, Accra, Ghana

**Keywords:** Asymptomatic infection, *Plasmodium*, Risk factors, Malaria burden, Ghana

## Abstract

**Background:**

Asymptomatic *Plasmodium* infections significantly drive malaria transmission and impact control and elimination strategies, but are largely uncharacterized. We investigated the prevalence and risk factors of asymptomatic malaria infections to inform malaria control strategies in Ghana.

**Method:**

Five cross-sectional surveys were conducted at the end of the peak transmission season (August–September) on 4892 school children aged between 6 and 14 years in two distinct ecological settings in Ghana between 2013 and 2017. The study sites were Begoro (forest ecology) and Cape Coast (coastal ecology). The children were screened for malaria parasites by microscopic examination of Giemsa-stained thin and thick blood films. Hemoglobin levels were measured using the Hemocue HB analyzer. In addition, height was measured and the height-for-age z-scores estimated from the reference population defined by WHO to determine children who were stunted. Proportions of categorical and means of continuous variables were compared using Chi-square test and Student’s t-test respectively, and multivariable logistic regression was done to assess risk factors associated with asymptomatic infections.

**Results:**

The overall prevalence of asymptomatic malaria in the school children was higher in Begoro compared to Cape Coast (27% (95% CI: 17, 24%) vs. 24% (95% CI: 17, 24%), *p* value = 0.04). The study recorded three species of *Plasmodium* (*Plasmodia falciparum, malariae*, and *ovale*) in both sites. *Plasmodium falciparum* was the predominant species, accounting for about 85% of infections in both study sites. The asymptomatic school children were more likely to be anaemic (OR = 2.01, *p* value< 0.001) and stunted in growth (OR = 1.46, *p* value< 0.001). Males carried more asymptomatic infection than females (OR = 1.18, *p* value = 0.015). School children aged 12–14 years had more asymptomatic infections than those aged 6–8 years (OR = 1.28, *p* value = 0.005).

**Conclusion:**

There is a considerable burden of asymptomatic malaria in the two regions of Ghana, which is associated with males, older children, anaemia, and stunted growth in children, and may have implications for malaria control and elimination strategies in Ghana.

## Background

Malaria is still one of the leading global public health concerns. Although there was a decline in malaria morbidity and mortality worldwide in 2018, sub-Saharan Africa saw an increase of about one million cases that same year [1]. Economic data from WHO malaria report shows that, in 2018, the WHO African Region spend nearly 3 quarter of the US$ 2.7 billion invested in malaria on case management and prevention [1] and cost up to 1.3% of GDP in Africa [2]. In addition, malaria is a major cause of absenteeism among school children in endemic countries [3–5].

Ghana is a malaria-endemic country and falls within the top 15 highest-burden countries [1]. In 2018, Ghana’s disease burden contributed to about 3% of the global malaria cases recorded [1] and 33% of all the national Out-Patient Department (OPD) cases recorded in 2017 [6]. Pregnant women and children under 5 years are the most affected by the disease because they have low immunity and malaria can cause anaemia in both groups resulting in debilitating outcomes like cerebral malaria and death [1].

*Plasmodium falciparum* infections may result in severe, uncomplicated or asymptomatic malaria. There is a wealth of studies and data on uncomplicated and severe malaria because they are the drivers of malaria-related morbidity and mortality respectively. Comparatively, asymptomatic malaria infection is understudied and remain a challenge to malaria control due to its effect on transmission dynamics [7].

Asymptomatic malaria, is defined as the presence of parasites in people and an absence of malaria-related symptoms such as temperature > 37.5 °C. Asymptomatic malaria occurs as a result of continuous exposure to malaria infections, leading to the acquisition of partial immunity against complications such as cerebral malaria and the accumulation of the “reservoir of infection” [8]. *P. falciparum* infection has been shown to persist asymptomatically in semi-immune individuals for more than 18 months especially in older children [7] making them important reservoirs for sustaining malaria transmission in regions of low and high malaria endemicity [9–15]. The asymptomatic reservoir contributes to gametocyte carriage (the stage of the life-cycle of the parasites that cause mosquito transmission) to drive and maintain transmission by the local mosquito vectors [7, 16]. Some findings suggest that asymptomatic carriers are more infectious than symptomatics [9, 17, 18] as they contribute to infectiousness for longer periods of time when they are not treated [19]. Aside from it being a reservoir for malaria transmission, asymptomatic carriage causes several other challenges including but not limited to anaemia, stunting, and cognitive impairment in school children [20, 21].

Like most malaria control programs, control efforts in Ghana gravitate towards a system, which identifies, treats, and reports people with malaria presenting at health care facilities giving little attention to asymptomatic cases in the community. However, through seasonal malaria chemoprevention and intermittent preventive treatments, a subset of asymptomatic infections is treated but not tracked. For successful malaria elimination, however, the full complement of the burden of infection, including the asymptomatic reservoir should be the focus. Thus, the asymptomatic carrier must be actively characterized [22].

To better understand the asymptomatic burden in Ghana, we leveraged repeated school-based cross-sectional surveys on the impact of parasite diversity on the evolution of drug resistance. We characterized the asymptomatic burden and its associated risk factors in two sites in Ghana with varying malaria endemicities. We show that the asymptomatic burden is significant in both sites and are influenced by risk factors such as age, anaemia, and stunted growth in children.

## Methods

### Study design, setting, population and ethics approval

Repeated cross-sectional surveys were conducted in Cape Coast and Begoro for five consecutive years (2013–2017). Cape Coast is in the coastal savanna ecological zone of Ghana where malaria transmission is low to moderate and perennial. Begoro is situated in the forest ecological zone of Ghana, where malaria transmission is high and perennial. The detailed description of these study sites has been described elsewhere [23]. The study participants included children aged between 6 and 14 years attending selected schools in the catchment areas of the study, whose parents/guardians gave informed consent to be part of the study and who also assented if aged 12 to 14. Three schools were randomly selected in each ecological zone and the children aged between 6 years and 14 years screened for malaria infection using microscopy. Children who were on malaria medication within 2 weeks prior to recruitment into the study and tested positive were excluded. The study was conducted following the latest Declaration of Helsinki and Good Clinical Practice (GCP). The protocol was approved by the ethics review board of Noguchi Memorial Institute for Medical Research, University of Ghana.

### Sample size and power

The study primarily compared the prevalence of asymptomatic malaria in the two study sites. A minimum sample size of 1933 per study site provides 80% power to determine as low as a 4% difference in asymptomatic malaria between study sites at a 95% confidence level and 5% precision.

### Sampling method

The list of all schools in the catchment areas of this study was obtained from the municipal education office and three schools randomly selected for each study site. All the school children aged 6 to 14 years from the selected schools, whose parents gave informed consent and assented if aged 12 to 14 were screened.

### Sample collection and diagnostic methods

Blood smears were prepared for each participant in the study. Two slides per participant were obtained: one with a thick smear and the other with both thick and thin smears. The thick smear slide was stained rapidly (10% Giemsa for 10–15 min) for initial screening, while the thick and thin smear slides were retained for subsequent quantification of the parasites if the participant tested positive. The parasite density was estimated, by counting the number of asexual parasites against 200 WBC with a hand tally counter. Parasite density was calculated as follows: Parasite density (per μl) = (number of parasites counted ÷ number of leukocytes counted) × 8000. Haemoglobin levels were determined for all patients using a portable HemoCue® Hb 301 Analyzer (HemoCue® AB, Ängelholm, Sweden). The body weight, height, and axillary temperature were also measured. Children who tested positive for malaria and had fever were treated with artesunate amodiaquine as recommended by the national malaria control program (NMPC).

### Definition of terms

- Asymptomatic malaria: the presence of parasites in peripheral thick blood smears, an axillary temperature < 37.5 °C, and an absence of malaria-related symptoms [7].
- Anaemia: Anaemia in children was defined as having haemoglobin (Hb) < 11 g/dL
- Stunting in children is defined as height-for-age z-score (HAZ) below negative two standard deviations from the median of the reference population, using the WHO Child Growth Standards

### Statistical analysis

The variables hemoglobin and height-for-age z-scores were categorized into binary variables. Height-for-age z-scores were categorized as “stunted” (z-score less than or equal to − 2 SD) and “not stunted” (z-score greater than − 2 SD), haemoglobin was categorized as anaemic (HB < 11 g/dl) and “non-anaemic” (HB ≥ 11 g/dl). All statistical analyses were performed using the statistical software STATA version 12. Continuous data were summarized into means and standard deviations (SD) (age, weight, height, parasite density, haemoglobin, and axillary temperature) and categorical data into proportions for descriptive analysis. Parasite density which is not normally distributed was log-transformed and the geometric mean calculated. Means were compared between study sites using Student’s t-test/Mann Whitney rank test, while proportions were compared using Pearson’s Chi-square tests/Fisher exact test. Trends of asymptomatic malaria over the study period were compared using the Cochrane Armitage test of trends. Univariate analysis to determine potential risk factors of asymptomatic malaria was performed using the Student’s t-test/Mann Whitney rank test for continuous variables and Pearson Chi-square test/Fisher exact test for categorical variables. The association between asymptomatic malaria and anaemia, stunting, gender, age, and study site was explored using multivariable logistic regression and the odds ratio and confidence intervals recorded. The significance level was set at *p* < 0.05 for all tests.

## Results

### Baseline characteristics of study participants and the prevalence of asymptomatic malaria

**Fig. 1:**
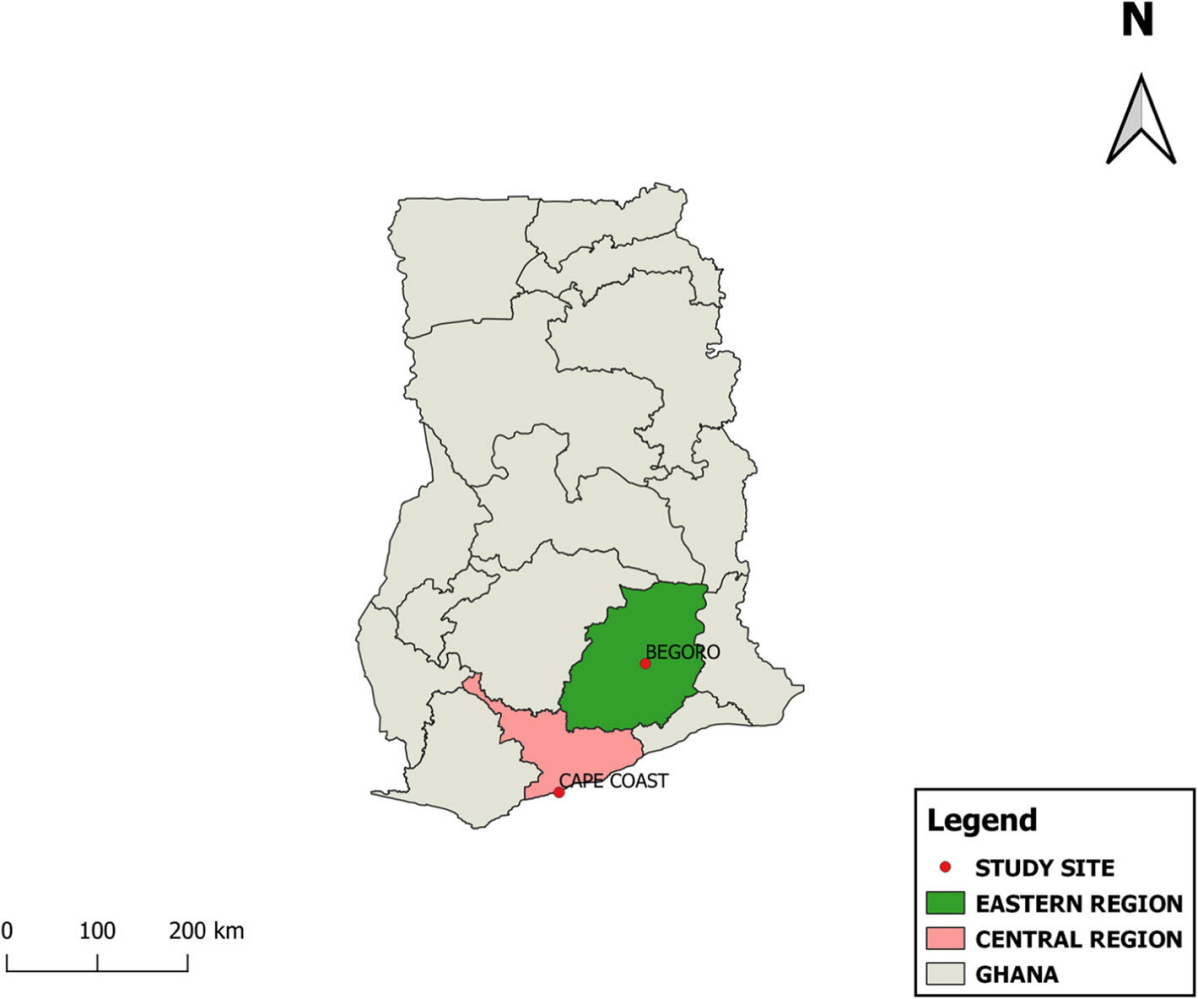
Map of Ghana showing the location of the two study sites considered in the study. Begorois considered a forest ecological zone where malaria is hyperendemic and has a high transmission intensity. Cape Coast, is considered a coastal savanna zone where malaria is also hyperendemic, but it has a low to moderate transmission intensity

**Fig. 2:**
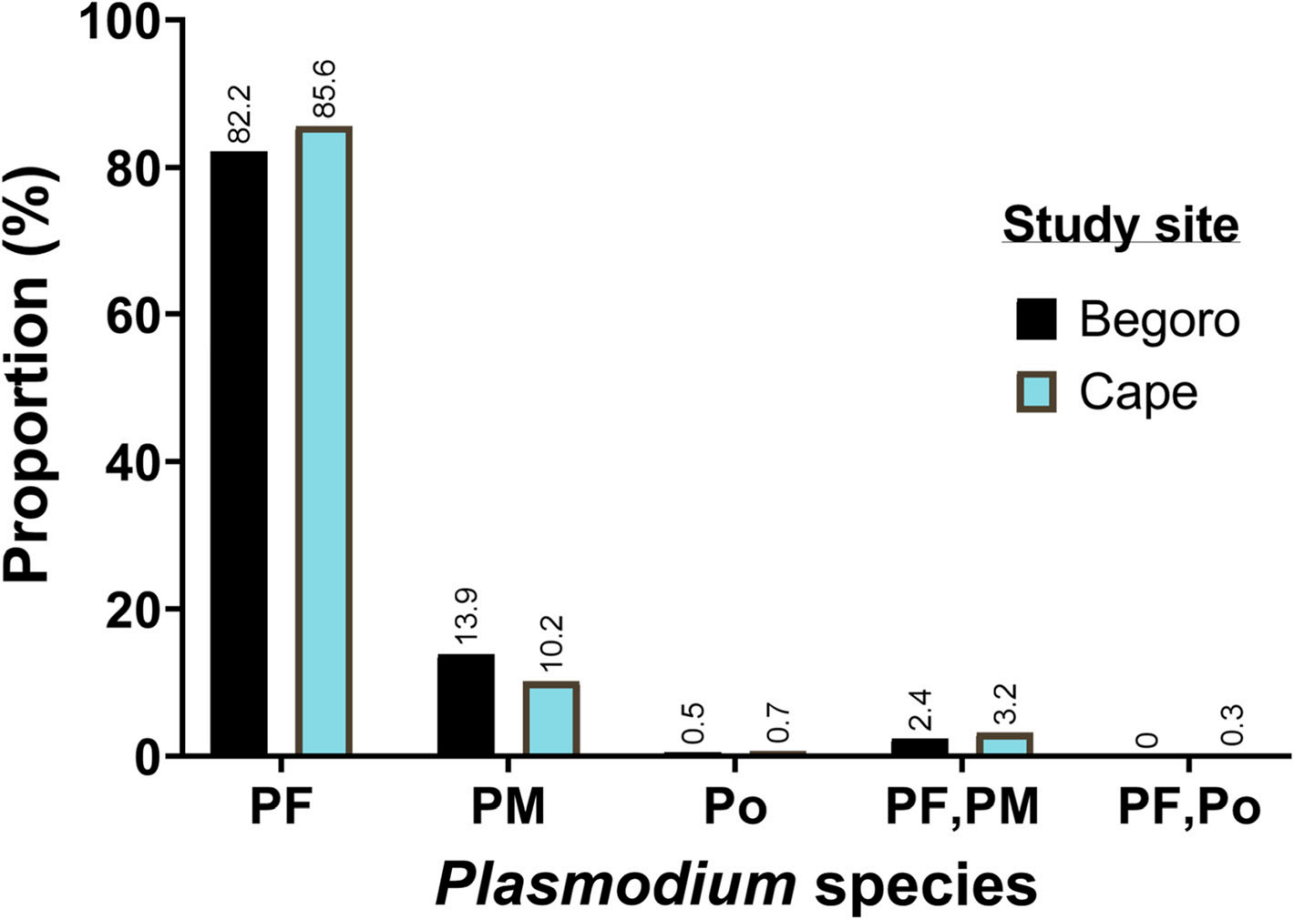
Proportion of *Plasmodium sp*. in two ecological zones in Ghana. PF = *Plasmodium falciparum*, PM = *Plasmodium malariae*, PO = *Plasmodium ovale*, PF, PM = *Plasmodium falciparum* and *malariae* mixed infection, and PF, PO = *Plasmodium falciparum* and *ovale* mix infection

A total of 4892 school children aged between 6 and 14 years, were screened; 2394 from Begoro and 2498 from Cape Coast over the five-year study period. Of these, 52.9% were females, 13.9% were anaemic, 11.1% had stunted growth, mean age was 10.3 years, and the geometric mean of parasite density was 289.4. Children in the Cape Coast municipal catchment area were more anaemic compared to those recruited from Begoro (17% vs 10% respectively, *p* value< 0.001). Also, the proportion of children from Cape Coast who were stunted was higher than observed in Begoro (14.5% vs 7.6% respectively, *p* value< 0.001). With the exception of 2014, the prevalence of asymptomatic malaria was always higher in Begoro compared to Cape Coast. The overall prevalence of asymptomatic malaria in Begoro was higher than in Cape Coast (27% vs 24%, *p* value = 0.04 Table 1), though there was no significant trend over the five-year period of the study. Three out of the five human *Plasmodium* species were found in both study sites (Fig. 1). These were *P. falciparum* (PF, 82 and 85.6% in Begoro and Cape Coast respectively), *P. malariae* (PM, 14 and 10% in Begoro and Cape Coast respectively), *P. ovale* (PO, < 1% in both Begoro and Cape Coast). Cape Coast had 3% mixed infections of PF, PM, and 0.3% of PF, PO, while, Begoro had 2% of PF, PM but no mixed infection of PF, PO. (Fig. 2 below).

**Table 1:** Baseline characteristics of study participants by ecological zones

Characteristics	Total	Ecological zone		
		Forest (FZ)	Coastal (CSZ)	*p* value
**Prevalence of malaria n/N (%)**				
2013	273/1037 (26.3)	127/478 (26.6)	146/559 (26.1)	0.869
2014	161/781 (20.6)	62/382 (16.2)	99/399 (24.8)	0.003
2015	261/935 (27.9)	142/452 (31.42)	119/483 (24.6)	0.021
2016	241/1027 (23.5)	127/525 (24.2)	114/502 (22.47)	0.576
2017	300/1112 (27.0)	178/557 (32.0)	122/555 (22.0)	< 0.001
Total	1236/4892 (25.3)	636/2394 (26.6)	600/2498 (24.0)	0.040
Mean age in years (SD)	10.3 (2.4)	10.3 (2.3)	10.3 (2.5)	0.5258
**Age group n (%) in years**				0.016
Aged 6 to 8 years	1247 (25.5)	602 (25.2)	645 (25.9)	
Aged 9 to 11 years	1862 (38.1)	959 (40.1)	903 (36.2)	
Aged 12 to 14 years	1779 (36.4)	833 (34.8)	946 (37.9)	
**Gender n (%)**				0.148
Female	2589 (52.9)	1292 (53.9)	1296 (51.9)	
Male	2303 (47.1)	1103 (46.1)	1202 (48.1)	
**Anaemia n/N (%)**				< 0.001
Normal	4160/4831 (86.1)	2139/2390 (89.5)	2021/2441 (82.8)	
Anaemic	671/4831 (13.9)	251/2390 (10.5)	420/2441 (17.2)	
**Stunted growth**				< 0.001
**Normal**	4349 (88.9)	2213 (92.4)	2136 (85.5)	
**Stunted**	545 (11.1)	182 (7.6)	363 (14.5)	
**Geometric mean parasitaemia/μL**	289.4	289.9	295.0	0.9331
Parasitaemia range	(13.79, 95,400)	(13.8, 65,910.5)	(8.0, 95,400)

### Determinants of asymptomatic malaria in school children

Table 2 shows the determinants of asymptomatic malaria in school-aged children in the two study sites in Ghana. All five risk factors considered: gender, age, anaemia, study site, and stunting were associated with asymptomatic malaria. Asymptomatic carriage was higher in males compared to females (28% vs. 23% *p* value = 0.001). In comparison with the other age groupings, the older children (aged between 12 and 14 years) were more asymptomatically infected (27% vs. 25 and 23%, *p* value = 0.019). The prevalence of asymptomatic malaria was higher in anaemic children compared to the non-anaemic children (38% vs. 23%, *p* value< 0.001). Prevalence of asymptomatic malaria was higher in children who were stunted (34%) compared to those who were not (34% vs. 24%, *p* value< 0.001). Children living in the Begoro catchment area were more likely to be asymptotic carriers than those living in Cape Coast (26.6% vs. 24%, *p* value< 0.04).

**Table 2:** Factors associated with asymptomatic malaria

Parameter	Asymptomatic malaria				*p* value
	No		Yes		
	N	%	N	%	
**Gender**					0.001
Male	1669	72.5	633	27.5	
Female	1986	76.7	603	23.3	
**Age group (years)**					0.019
6 to 8	962	77.2	284	22.8	
9 to 11	1396	75.0	466	25.0	
12 to 14	1293	72.7	485	27.3	
**Anaemia**					< 0.001
Non anaemic (≥11)	3193	76.8	965	23.2	
Anaemic (< 11)	414	61.7	257	38.3	
**Stunted growth**					< 0.001
Normal	3295	75.8	1053	24.2	
Stunted	361	66.2	184	33.8	
**Study site**					
Begoro	1758	73.4	636	26.6	0.04
Cape Coast	1898	76.0	600	24.0	

### Multivariable analysis of potential risk factors of asymptomatic malaria

A logistic regression model was used to explore the associations between the potential risk factors and asymptomatic malaria. After adjusting for confounders (age, stunted growth, anaemia and study site), being male increased the odds of asymptomatic malaria by 18% (OR = 1.18, 95% CI = 1.03, 1.35, *p* value = 0.015). The odds of asymptomatic malaria in the 12–14 years age group was 1.28 times higher than that in the 6–8 years age group (OR = 1.28, 95%CI = 1.08, 1.53, *p* value = 0.005). Anaemic children were twice more likely to carry asymptomatic infections (OR = 2.01, 95%CI = 1.77, 2.51, *p* value< 0.001) than the non-anaemic children and stunted growth increased the odds of carrying asymptomatic parasites by 46% (OR = 1.46, 95%CI = 1.19, 1.80, *p* value< 0.001). In addition, the study showed an increased odd of 26% asymptomatic carriage in Begoro (forest ecological zone) compared to Cape Coast (in the coastal savanna zone) after adjusting for age, sex, anaemia and stunted growth (OR = 1.26, 95% CI = 1.10, 1.44, *p* value = 0.001) (Table 3).

**Table 3:** Some risk factors associated with asymptomatic malaria

Parameter		Asymptomatic malaria			
	Exposed group	Unadjusted OR (95% CI)	*p* value	Adjusted OR (95% CI)	*p* value
Gender	Males	1.24 (1.10, 1.42)	0.001	1.18 (1.03, 1.35)	0.015
Age group (years)	9 to 11	1.13 (0.96, 1.34)	0.154	1.11 (0.93, 1.32)	0.2235
	12 to 14	1.27 (1.07, 1.50)	0.005	1.28 (1.08, 1.53)	0.005
Anaemia	Anaemic	2.05 (1.73, 2.44)	< 0.0001	2.01 (1.77, 2.51)	< 0.001
Stunted growth	Stunted	1.59 (1.32, 1.93)	< 0.0001	1.46 (1.19, 1.80)	< 0.001
Study site	Begoro	1.14 (1.01, 1.30)	0.04	1.26 (1.10, 1.44)	0.001

## Discussion

With the clarion call for malaria elimination, attention has been drawn to the asymptomatic reservoir of infection as it significantly contributes to *Plasmodium* transmission. Thus, recent epidemiological studies in most malaria-endemic countries are not only focusing on symptomatic malaria for disease morbidity and mortality rates but also the asymptomatic infection rate to fully define the disease burden during malaria control and elimination interventions.

Our repeated cross-sectional surveys estimated the prevalence of the asymptomatic infections in catchment areas of two ecological zones in Ghana and also identified associated risk factors of asymptomatic malaria. The study revealed a high asymptomatic burden (~ 20%) in the two catchment areas studied, where most of the infections were *P. falciparum.* A few infections were (*P. malariae, P. ovale,* with mixed infection *P. falciparum* + *P. malariae* and *P. falciparum* + *P. ovale*). As expected, asymptomatic malaria was higher in Begoro, within the forest ecological niche compared to Cape Coast in the coastal region of Ghana and was associated with age, gender, anaemia and stunted growth in school children. The outcomes of this study highlight the need to expand control interventions and educational campaigns for asymptomatic malaria in Ghana with particular focus on males and older children.

The variation in asymptomatic carriage in these different ecological zones is consistent with transmission intensity in these two sites. The increased odds of asymptomatic malaria in the forest ecological zone may be due to more favorable climatic conditions such as temperature, abundant rainfall and the vegetative cover, resulting in an abundance of breeding sites for the mosquito vector in these areas. These variations observed in transmission between communities reflect the importance of micro-ecological factors and transmission in the areas studied [24]. The bulk of malaria cases in sub-Saharan Africa are caused by *Plasmodium falciparum*, with other *Plasmodium* species having a low but underestimated prevalence [1, 25]. The distribution of *Plasmodium* species in this study is consistent with other studies conducted in Ghana. *P. falciparum* is the most common malaria-causing species in Ghana, followed by *P. malariae* and *P. ovale* [25–27].

The prevalence of asymptomatic infections among children was more predominant in males than females. This disparity may come as a result of gender roles with regards to division of labour, hormonal or host genetic factors [28, 29], and behavioral factors like leisure activities and sleeping patterns [30]. Males tend to stay late outdoors and without proper protective clothing and they also have the tendency to sleep outdoors or not sleep under a mosquito net. It has been suggested that males usually have lower immune response than females [31]. The immunological differences between males and females, linked to circulating steroid hormones such as testosterone, oestradiol, and progesterone may explain the increased parasitism in males [31]. Not only do host hormones influence infection responses, but parasites in their hosts can also generate and modify hormone concentrations [32]. Some of these gender differences that affect disease outcomes includes access to immunization, nutritional status, access to, and use of preventive and curative health care, including differences in the speed with which males and females get treatment outside the home [30]. Understanding how gendered patterns of behavior influence exposure to mosquitoes can therefore assist in developing more-effective recommendations for preventing malaria infection especially in males.

In this study, children aged between 12 and 14 years were 21% more asymptomatic than children aged between 6 and 8 years. This was consistent with findings from other studies conducted in Ghana [33–35]. Age has been shown to be associated with malaria in general and studies have reported its association with asymptomatic malaria carriage [34]. An increase in age is positively associated with protective immunity to malaria in endemic settings where older children and adults have acquired partial immunity and are more likely to carry asymptomatic infections [34, 36].

Our study recorded a doubled risk of anaemia in children who had asymptomatic infections. Malaria-associated anaemia has been well documented, however, few studies have looked at the impact of asymptomatic malaria on anaemia in healthy children in schools. In general, malaria contributes to the loss of iron from lysed cells through excretion, impairment of intestinal absorption of ingested iron, the release of storage iron from hepatocytes, and recycling of iron that is derived from phagocytosis of senescent or parasitized erythrocytes by macrophages [37]. However, asymptomatic lower-density parasitemia may also contribute to anaemia, particularly if the parasitemia persists for prolonged periods due to lack of, or ineffective treatment [38, 39]. In addition, school children living in the Cape coast catchment area were more anaemic than their counterparts living in Begoro. Although our study was not designed to obtain information on diet, the anecdotal evidence indicates that the vegetation of Begoro allows for an abundant propagation of iron rich green leafy vegetables as staple for the community including our study catchment area in comparison with Cape Coast where the vegetation is mostly mangrove with salty patches.

Our study revealed that stunting was associated with asymptomatic malaria though a similar study conducted in another forest region of Ghana did not show an association between stunting and asymptomatic malaria [40]. The inconsistency in the results may be due to smaller sample size in the other study. Evidence of an association between malaria risk and anthropometric indicators such as stunting remains inconclusive [41]. While some studies have reported that stunting is associated with a higher risk of asymptomatic malaria as reported in this study [42, 43], others have suggested a protective effect [20, 44–47]. For instance, Mitangala and colleagues in 2013 showed that severely stunted children were at a lower risk of high-level malaria [46]. Some studies also suggest no association between anthropometric indicators and asymptomatic malaria risk [40, 48, 49]. How malnutrition influences malaria morbidity and mortality is debatable. Some studies suggest that malnourished children seem to be more susceptible to malaria parasite carriage because of decreased immune system functioning [50]. This complex relationship may be influenced by confounders like socio-economic factors such as poverty, level of literacy of parent/guardian, common diet of community etc. [51].

This study had a few limitations. The study might have under-reported the prevalence of asymptomatic malaria because microscopic detection of parasites as used in the study has low sensitivity compared to polymerase chain reaction (PCR), although it is the gold standard for diagnosing malaria in endemic countries. Evidence of under-reporting of malaria cases by microscopy has been shown [52, 53] compared to PCR. Microscopy reported a false negative diagnosis of 19.4% [52]. Also, the study did not consider household factors and the use of insecticide treated bed nets, which are also risk factors of malaria.

## Conclusion

The prevalence of asymptomatic malaria was high in both study sites in Ghana. Asymptomatic malaria among the school children was age-dependent with a higher risk in male children. A significant association was indicated between asymptomatic malaria and the risk of anaemia and stunting. Thus, malaria control programs should also focus on asymptomatic malaria as a means to monitor the impact of control interventions and to reduce malaria morbidity and mortality.

## Data Availability

The dataset used and/or analysed during the current study is available from the corresponding author on reasonable request.

## Abbreviations

GCP: Good Clinical Practice
HAZ: Height-for-Age Z scores
OPD: Out-Patient Department
NMPC: National Malaria Control Program
PF: *Plasmodium falciparum*
PM: *Plasmodium malariae*
PO: *Plasmodium ovale*
WHO: World Health Organization
